# Supplementary material for: tRNA epitranscriptomic alterations associated with opioid-induced reward-seeking and long-term opioid withdrawal in male mice
Source: Neuropsychopharmacology. 2024 Feb 8;49(8):1276–84. doi: 10.1038/s41386-024-01813-6 (PMC11224224; doi:10.1038/s41386-024-01813-6)
Supplement: Supplementary file 1 — Supplementary Material [file 41386_2024_1813_MOESM1_ESM.docx]

**Supplementary Information**

Blaze, Browne et al.

**tRNA epitranscriptomic alterations associated with opioid-induced reward-seeking and long-term opioid withdrawal in male mice**

**Supplementary Figures**

**
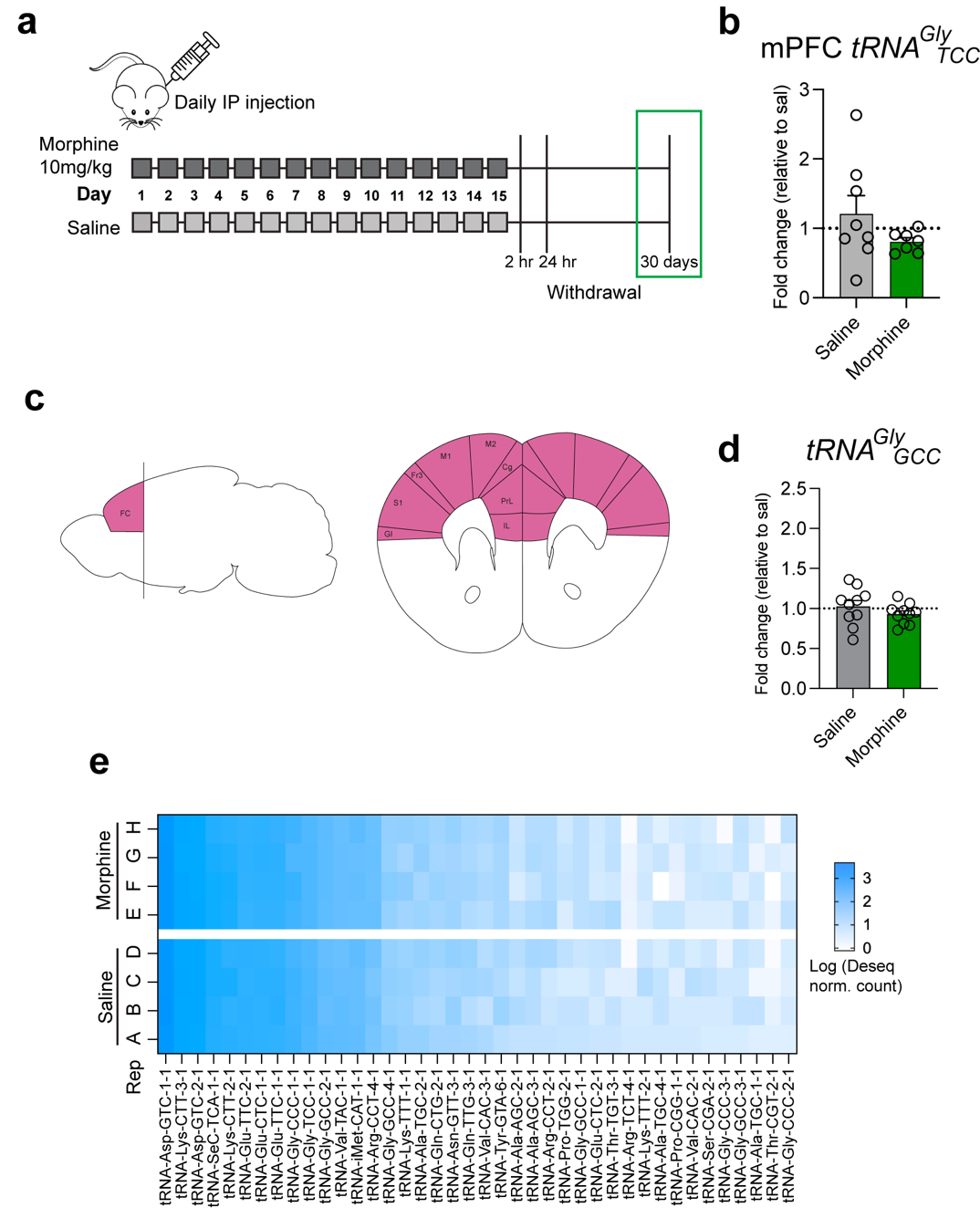
**

**Supplementary Figure 1. a)** After 30 days of withdrawal, mPFC was collected and **b)** showed no change in expression of another tRNA^Gly^ isoacceptor, tRNA^Gly^_TCC._ **c)** Brain atlas localization of tissue taken for frontal cortex molecular assays. **d)** After 30 days withdrawal, frontal cortex showed no change in tRNA^Gly^_GCC_ expression (n=10/group). **e)** Heatmap shows top 40 isodecoders that were most highly and reliably expressed in all samples using Deseq normalized counts.

**
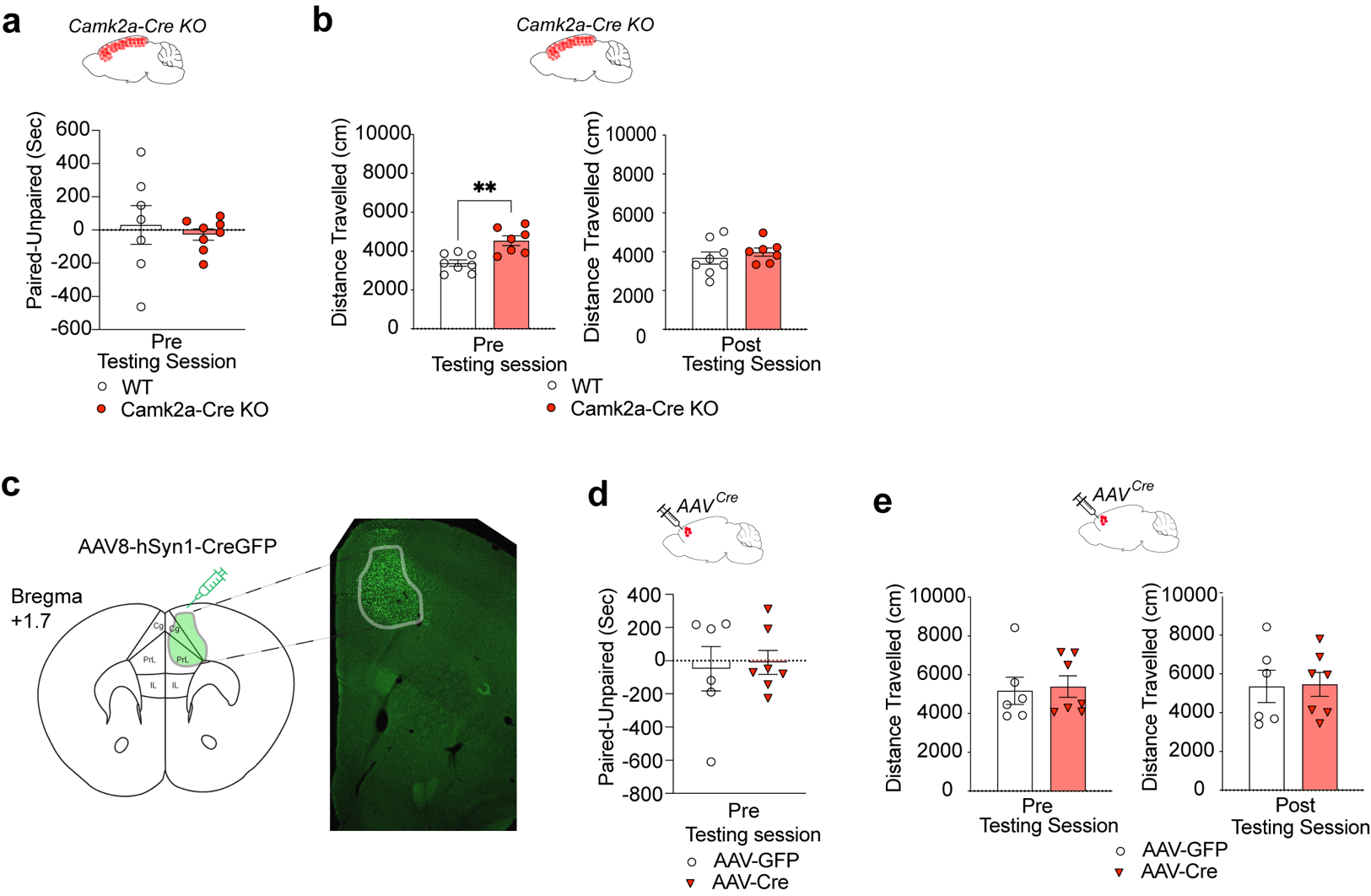
Supplementary Figure 2.** Additional behavioral and histological data for Nsun2 conditional knockout mice. Time in paired and unpaired side of chamber during CPP in pre-test for **(a)** Camk2a-Cre NSUN2 KO mice and **(c,d)** PFC AAV-Cre NSUN2 KO mice showing lack of group difference in preference. Activity measured as distance traveled (cm) in CPP chambers during pre-test and post-test phases for **(b)** Camk2a-Cre NSUN2 KO mice and **(c,e)** PFC AAV-Cre NSUN2 KO mice. **(c)** Representative image of PFC injection of AAV8-hSyn1-CreGFP, showing GFP immunofluorescence in prelimbic and cingulate cortex. (t-test **p<0.01)**.**

**Supplementary Table 1.** Site-specific statistical analysis of bisulfite tRNA sequencing
